# Study on public perceptions and protective behaviors regarding Lyme disease among the general public in the Netherlands: implications for prevention programs

**DOI:** 10.1186/1471-2458-13-225

**Published:** 2013-03-15

**Authors:** Desirée Jacqueline Mathieu Angélique Beaujean, Marloes Bults, Jim Everardus van Steenbergen, Hélene Antoine Claire Marie Voeten

**Affiliations:** 1 National Institute of Public Health and the Environment, Centre for Infectious Disease Control, P.O. Box 1, Bilthoven 3720 BA, The Netherlands; 2 Municipal Public Health Service Rotterdam-Rijnmond, P.O. Box 70032, Rotterdam, 3000 LP, The Netherlands; 3 Department of Public Health, Erasmus MC and University Medical Center Rotterdam, P.O. Box 2040, Rotterdam, 3000 CA, The Netherlands

**Keywords:** Perceptions, Lyme disease, Ticks, Tick bites, General public, Prevention, Protective behavior, Knowledge, Concern

## Abstract

**Background:**

Lyme disease (LD) is the most common tick-borne disease in the United States and in Europe. The aim of this study was to examine knowledge, perceived risk, feelings of anxiety, and behavioral responses of the general public in relation to tick bites and LD in the Netherlands.

**Methods:**

From a representative Internet panel a random sample was drawn of 550 panel members aged 18 years and older (8-15 November 2010) who were invited to complete an online questionnaire.

**Results:**

Response rate (362/550, 66%). This study demonstrates that knowledge, level of concern, and perceived efficacy are the main determinants of preventive behavior. 35% (n = 125/362) of the respondents reported a good general knowledge of LD. While 95% (n = 344/362) perceived LD as severe or very severe, the minority (n = 130/362, 36%) perceived their risk of LD to be low. Respondents were more likely to check their skin after being outdoors and remove ticks if necessary, than to wear protective clothing and/or use insect repellent skin products. The percentage of respondents taking preventive measures ranged from 6% for using insect repellent skin products, to 37% for wearing protective clothing. History of tick bites, higher levels of knowledge and moderate/high levels of worry were significant predictors of checking the skin. Significant predictors of wearing protective clothing were being unemployed/retired, higher knowledge levels, higher levels of worry about LD and higher levels of perceived efficacy of wearing protective clothing.

**Conclusions:**

Prevention programs targeting tick bites and LD should aim at influencing people’s perceptions and increasing their knowledge and perceived efficacy of protective behavior. This can be done by strengthening motivators (e.g. knowledge, concern about LD, perceived efficacy of wearing protective clothing) and removing barriers (e.g. low perceived personal risk, not knowing how to recognize a tick). The challenge is to take our study findings and translate them into appropriate prevention strategies.

## Background

Lyme disease (LD) is the most common tick-borne disease in the United States and in Europe. In the Netherlands, the number of General Practitioner (GP) consultations for tick bites has increased from 191 per 100,000 in 1994 to 564 per 100,000 in 2009 [1]. In 1994, the incidence of patients visiting the GP for Erythema migrans (EM, an asociated symptom) was estimated at 39 per 100,000 inhabitants. This number increased to 134 per 100,000 in 2009 [1]. Similar trends have been observed in other European countries [2]. The emergence of Lyme borreliosis may have been partly caused by an increased awareness among citizens and medical personnel, and changes in pathogen and vector populations [3].

Transmission of LD requires the presence in the area of: (1) the spirochete *Borrelia burgdorferii* sensu lato (in Europe) in an animal reservoir that is capable of transmitting the spirochetes to feeding ticks; (2) the vector, in European ticks belonging to the *Ixodes ricinus* group; and (3) susceptible hosts, including humans. Forested areas and recreational sites such as parks and gardens are associated with a higher risk of tick bites [4].

In humans LD develops in three stages. In the first stage (the early localized infection), people may experience fever, headache, fatigue and depression. The most commonly recognized symptom at this stage (occurring in about 90% of patients) is a circular red skin rash around the place of the tick bite: erythema migrans. Antibiotics administered at this stage will prevent further stages developing. Untreated, the infection may spread from the site of the bite to other parts of the body, producing an array of distinct symptoms that may come and go, including: additional EM lesions in other areas of the body, facial or Bell’s palsy (loss of muscle tone on one or both sides of the face), severe headaches and neck stiffness due to meningitis (inflammation of the spinal cord), pain and swelling in the large joints (such as knees), shooting pains that may interfere with sleep, heart palpitations and dizziness due to changes in the heartbeat. This is called the second stage (early disseminated stage). Many of these symptoms will resolve over a period of weeks to months, even without treatment. However, a lack of treatment can result in additional complications. Approximately 60% of patients with an untreated infection may begin to have intermittent bouts of arthritis, with severe joint pain and swelling. Without treatment, LD enters the third stage after several months. During this stage, the patients develop chronic symptoms that can affect a wide range of body parts, including the brain, nerves, joints and the heart [5].

Recommendations for first line treatment from most European countries and the USA specify doxycycline or amoxicillin, with minor differences in doses and treatment duration. Both agents have proven efficacy, but a small proportion of patients have persistent symptoms following appropriate treatment for LD [6]. Because no vaccine is available and effective measures for controlling tick populations are still in the experimental phase or insufficient, health education is considered the most important approach for preventing LD. The National Institute of Public Health and The Environment (RIVM) provides information on public health topics to professionals and to the general public. This includes a national guideline on the prevention and control of LD for professionals [7] and several brochures for the general public. In the Netherlands, the local public health services are responsible for sending out the guideline and brochures to professionals (e.g. GP’s, camping holders) and to the public.

Preventive strategies include the avoidance of tick-infested areas, the use of protective clothing (e.g. wearing long-sleeved shirts and long trouserpants, which reduce the area of exposed skin), routine body checks for ticks after being outdoors, and the use of tick repellents on either the skin or clothing.

Despite the availability of Lyme prevention advice, public compliance with the LD-guidelines could be improved [8]. Surveillance of perceptions and behavioral responses of the general public to ticks and LD is required for improving health risk communication and achieving successful changes in public behavior [9-13].

However, in the Netherlands very little is known about the perception and protective behavior of people in relation to the prevention of tick bites and LD [14]. In 2010 Maat and Konings found in their study among 600 residents in the Southwest of the Netherlands that many respondents lacked skills for recognizing and removing ticks, underestimated their personal risk for tick bites and found protective measures exaggerated [15]. They concluded that new prevention strategies should focus on self-efficacy, risk perception, and the presentation of alternative measures, e.g. skin check instead of wearing protective clothing. In the present study our aim was to examine the knowledge, perceived risk, feelings of anxiety, and behavioral responses of the Dutch general public related to ticks and LD. The questionnaire was based on an integrated model to explain health behavior, including constructs from the Protection Motivation Theory and the Health Belief Model [16,17]. Protection Motivation theory has been used as a model for predicting health behavior. A threat appraisal is formed by an individual based on their perceived likelihood of a particular event occurring and their perceived severity of the event. The way in which an individual chooses to respond to a threatening situation is termed their coping appraisal, and is based on both the belief that uptake of a recommended behavior will resolve the threat (response efficacy), and an individual’s belief in his/her own ability to effectively perform the behavior (self-efficacy).

In the Health Belief Model, perceptions of the individual are at the core of the model, which posits that the beliefs of individuals about their own susceptibility to a health threat, their perceptions about the severity of that threat, and their perceptions about the benefits and barriers associated with a particular protective action, will determine whether or not they adopt that action. Extensions to the model suggest that the ‘self-efficacy’ (their belief in their own ability to perform a given behavior) of an individual also plays a strong role in determining whether a behavior is adopted, as does the existence of cues to action that prompt or remind someone to engage in a particlar behavior.

The results will act as a guide for further development of effective LD prevention programs by identifying those measures most likely to be adopted by the general public in the Netherlands.

## Methods

### Participants

For this study a representative Internet panel was used, named the Flycatcher panel (http://www.flycatcher.eu). This panel consists of members from the Dutch general public who volunteer to participate in online questionnaire surveys. The panel consists of 20.000 members with a representative distribution of demographic variables (gender, age, region, and level of education) for the general Dutch population. The panel meets high quality requirements and is ISO-certified. A random sample of 550 panel members aged 18 years and older was drawn. Panel members in this selection were invited to participate in this study by sending an email with a linking to an online questionnaire. The survey remained online from 8 to 15 November 2010. No reminders were sent out. To motivate enrollment, participants received 1.50 Euro in credits for completion of the survey, which could be exchanged for gift vouchers.

The nature of this general Internet-based survey among healthy volunteers from the general population does not require formal medical ethical approval according to Dutch law [18].

### Questionnaire

The online questionnaire (45 questions) was developed based on an existing questionnaire used in studies on risk perception and precautionary behaviors of the general public during the 2009-H1N1 flu pandemic and during outbreaks of SARS and avian Influenza [19-21]. To examine people’s perceptions of LD and the preventive measures and the predictors of protective behavior we included the following constructs: knowledge, perceived severity of and vulnerability to LD, feelings of concern, perceived efficacy of preventive measures, a person’s ability (self-efficacy) and intention to take measures, actual behaviors, and main motivators and barriers taking measures. Knowledge was examined according to five statements concerning modes of transmission, incidence of tick bites and preventive measures. For knowledge, a summary score was created based on the number of correct answers and dichotomized as 0 (≤3 items correct) or 1 (4-5 items correct). Perceived severity was measured by two items, namely “How serious do you think Lyme disease is?” and “How awful would it be if you were diagnosed with LD in the next 12 months?”. Perceived vulnerability was also measured by two items, namely “Do you think that you are susceptible getting LD, if you don’t take preventive measures?” and “How likely is it that you will be diagnosed with LD in the next 12 months?”. Feelings of concern were measured by asking respondents “How worried are you about getting LD?”.

Participants were asked about four preventive measures for LD, namely: ‘wearing protective clothing that cover the body (i.e. long trousers/sleeves)’; ‘using insect repellent skin products’; ‘checking the skin after being outdoors’ and ‘removing ticks from the skin’. Perceived efficacy of these preventive measures was formulated as “Do you think [*measure X*] helps to prevent Lyme disease?”; self-efficacy as “Do you think you are able to perform [*measure X*]?”; intention as “Do you intend to perform [*measure X*]?’; and behavior as “How often do you perform [*measure X*]?”. People who indicated that they had never performed a certain preventive behavior were asked to tick a maximum of three barriers from a list of possible barriers (to which they could add their own barrier); people who indicated that they had sometimes/often/always performed a certain behavior were asked to tick a maximum of three motivators from a list of possible motivators (to which they could and their own motivator). Barriers and motivators were generated from the literature (including unpublished/grey literature) [15,22-24].

### Analysis

Descriptive statistics were performed. Due to skewed distributions, and to aid interpretation of the results, constructs were dichotomized into low and high scores (see Table 1), and the Chi-squared test was used to test the statistical significance of group differences (gender, age, educational level, and employment status) regarding knowledge, perceived severity, perceived vulnerability, feelings of concern, perceived efficacy of preventive measures, perceived self-efficacy, intention and preventive measures taken.

**Table 1:** Perceptions regarding Lyme disease and preventive measures (n = 362)

	**Gender**			**Age**				**Education**				**Employment status**			**Overall**	
	**Male (%)**	**Female (%)**	**p-Value**	**18-29 (%)**	**30-49 (%)**	**≥50 (%)**	**p-Value**	**Low (%)**	**Middle (%)**	**High (%)**	**p-Value**	**Employed (%)**	**Unemployed/retired (%)**	**p-Value**	**%**	**n/ntot**
**Knowledge (5 statements)**^**2**^																
4 or 5 correct	31	38	ns	31	35	35	ns	33	35	36	ns	32	38	ns	35	(125/362)
**Perceived severity (scale 1-5; not at all – very severe/awful)**																
Seriousness of LD																
score 4-5 (severe/very severe)	93	97	0.047	96	97	94	ns	94	97	93	ns	95	95	ns	95	(344/362)
Severity being diagnosed with LD in the next 12 months																
score 4-5 (awful/very awful)	94	97	ns	100	96	94	ns	96	96	95	ns	95	95	ns	95	(345/362)
**Perceived vulnerability (scale 1-5; not at all – very susceptible/likely)**																
Susceptibility to LD																
score 4-5 (quite susceptible/very susceptible)	38	34	ns	43	33	36	ns	31	35	44	ns	38	33	ns	36	(130/362)
Likelihood being diagnosed with LD in the next 12 months																
score 4-5 (likely/very likely)	4	4	ns	2	4	5	ns	3	7	2	ns	5	3	ns	4	(15/362)
**Feelings of concern (scale 1-5; not at all – very worried)**																
score 3-5 (a bit worried/worried/very worried)	51	55	ns	37	60	54	0.03	53	55	52	ns	56	50	ns	53	(193/362)
**Perceived efficacy (scale 1-5; certainly not – certainly)**																
Wear protective clothing (score 4-5)	92	89	ns	78	91	94	0.005	90	91	90	ns	87	95	0.01	90	(327/362)
Check your skin after being outdoors (score 4-5)	79	87	0.04	88	81	84	ns	82	85	84	ns	82	85	ns	83	(302/362)
Remove tick from your skin (score 4-5)	73	71	ns	73	69	74	ns	68	79	67	ns	72	72	ns	72	(260/362)
Use insect repellent skin products (score 4-5)	31	37	ns	31	39	31	ns	31	39	31	ns	36	31	ns	34	(123/362)
**Perceived self-efficacy (scale 1-5; certainly not – certainly)**																
Wear protective clothing (score 4-5)	40	37	ns	35	31	45	0.04	44	33	39	ns	33	47	0.005	39	(140/362)
Check your skin after being outdoors (score 4-5)	59	69	0.03	69	62	64	ns	67	68	54	ns	63	65	ns	64	(232/362)
Remove tick from your skin (score 4-5)	77	72	ns	78	71	75	ns	69	77	79	ns	74	75	ns	74	(269/362)
Use insect repellent skin products (score 4-5)	21	38	<0.001	26	33	29	ns	36	29	21	0.045	28	32	ns	30	(108/362)
**Intention (scale 1-5; certainly not – certainly)**																
Wear protective clothing (score 4-5)	39	38	ns	24	29	49	<0.001	46	37	30	0.04	28	52	<0.001	38	(139/362)
Check your skin after being outdoors (score 4-5)	48	58	ns	53	54	52	ns	53	57	47	ns	53	54	ns	53	(192/362)
Remove tick from your skin (score 4-5)	92	93	ns	88	91	96	ns	93	92	93	ns	91	95	ns	93	(336/362)
Use insect repellent skin products (score 4-5)	17	27	0.03	20	25	21	ns	26	23	15	ns	22	23	ns	22	(80/362)
**Behavior (scale 1-4; never - always)**																
Wear protective clothing (score 3-4)	39	36	ns	28	33	43	ns	37	38	36	ns	31	46	0.002	37	(135/362)
Check your skin after being outdoors (score 3-4)	30	34	ns	39	31	30	ns	27	38	29	ns	32	31	ns	32	(115/362)
Use insect repellent skin products (score 3-4)	7	6	ns	6	8	5	ns	7	4	8	ns	7	5	ns	6	(22/362)

LD = Lyme Disease; ^2^ The five statements were: people can get Lyme disease after a tick bite; during the summer, the chance on tick bites is higher compared to the winter; ticks mostly fall out of trees; about 1 in 15 people in the Netherlands are yearly bitten by ticks; using repellent skin products can protect against tick bites.

Univariate and multivariate logistic regression analyses were performed to identify factors significantly associated with 1) wearing protective clothing and 2) checking skin after being outdoors. For the multivariate regression analyses, a backward ‘elimination’ procedure was employed, starting with all potential independent variables (all variables with a p-value <0.1 in the univariate analysis), and then with the least significant variable removed at each step (the one with the highest p-value), until only statistically significant predictors (p < 0.05) remained.

## Results

A total of 550 panel members were invited to participate in this study, of whom 362 completed the questionnaire (response rate 66%). Of the 362 respondents, 51% was female (data not shown). The age varied from 18-29 years (14%), 30-49 years (35%) and ≥50 years (51%). Thirty-seven percent had a low educational level (i.e. primary education, lower general or lower vocational education or less), 38% an intermediate (i.e. secondary general or vocational education), and 25% a higher education (i.e. higher professionals education or university). More than half of the respondents were employed, and in 36% of the households there were one or more children. The overall majority were of Dutch origin. Most respondents lived in the middle (45%) and the south (46%) of the Netherlands. Twelve percent of the respondents had had a tick bite once and 9% had repeatedly had tick bites in the Netherlands. Around half of the respondents were regularly (every week/once a month) physically active in a garden, 37% visited the woods regularly and 21% visited open areas regularly.

### Knowledge

Of the respondents, 125 (35%) answered at least 4 out of 5 knowledge statements correctly (Table 1) and were categorized as having “good general knowledge”. The statements “people can get LD after a tick bite” and “during the summer, the chance of tick bites is higher compared to the winter” were correctly answered by the majority of the respondents (98% and 90% respectively; data not shown). Remarkably, only 22% were aware that “using repellent skin products can protect against tick bites”.

### Perceived severity and vulnerability

Of the respondents, 95% perceived LD to be severe or very severe, significantly more often by female (n = 179, 97%) than by male respondents (n = 165, 93%), (p = 0.047). Equally, (n = 345), 95% reported that it would be awful or very awful if they were diagnosed with LD in the next 12 months, while 36% (n = 130) perceived themselves as quite/very susceptible, and only 4% believed it was likely or very likely that they would be diagnosed with LD in the next 12 months. Around half of the respondents reported feelings of concern about getting LD, significantly less frequently in respondents aged 18-29 years (n = 19, 37%) compared to the other age groups (n = 176, 57%) (p = 0.03).

### Self-efficacy, response efficacy and intention

Wearing protective clothing and checking the skin after being outdoors were perceived as the most effective measures for preventing tick bites. Respondents aged 18-29 and those employed reported high perceived efficacy of wearing protective clothing less often (p = 0.005 and p = 0.01 respectively), whereas male respondents reported high perceived efficacy of checking the skin (p = 0.04) less often. Almost three quarters reported high perceived efficacy of removing a tick from their skin, and only a minority of the respondents (34%) reported high perceived efficacy of using repellent skin products. The majority of respondents reported high perceived self-efficacy for removing ticks from their skin (74%) and checking their skin after being outdoors (64%). Female respondents (p = 0.03) reported high perceived self-efficacy of checking their skin after being outdoors more often. Only 39% reported high perceived self-efficacy of wearing protective clothing, which was more often reported by respondents aged 50 years and older and those unemployed/retired (resp. p = 0.04 and p = 0.005 respectively). High perceived self-efficacy of using repellent skin products was reported by 30% of the respondents. Female respondents (p < 0.001) and lower educated (p = 0.045) reported high perceived self-efficacy for using repellent skin products more often.

The overall majority (93%) reported a high intention to remove a tick from their skin, if necessary, and 53% reported high intention for checking their skin. High intention to wear protective clothing was observed in 38% of the respondents. Respondents aged 50 years and older (p < 0.001), those with a lower education (p = 0.04) and unemployed/retired (p < 0.001) reported high intention to wear protective clothing more often. Twenty-two percent of the respondents reported high intention for using insect repellent skin products. Female respondents reported high intention to use repellent skin products more often (p = 0.03).

### Preventive measures taken

Thirty-seven percent of the respondents reported wearing protective clothing when going into nature areas (30% who reported this behavior often and 8% always; data not shown). Unemployed/retired respondents reported to wear protective clothing more often compared to those employed (p = 0.002). Thirty-two percent of the respondents reported checking their skin after they had been outdoors (21% often and 11% always). A minority (6%) reported to use insect repellent skin products (5% often and 1% always).

### Determinants of preventive behavior

Univariate and multivariate logistic regression analyses were performed to identify factors significantly associated with 1) wearing protective clothing and 2) checking the skin after being outdoors. Table 2 shows significant predictors of wearing protective clothing that were selected using the univariate logistic regression. From the multivariate logistic regression analysis, predictors of wearing protective clothing were: being unemployed/retired (OR 1.96; 95% CI 1.24-3.08), higher knowledge levels (OR 1.69; 95% CI 1.07-2.68), higher levels of concern about LD (OR 2.22; 95% CI 1.41-3.51) and higher levels of perceived efficacy of wearing protective clothing (OR 2.97; 95% CI 1.17-7.54). Table 3 shows significant predictors of checking the skin for the presence of ticks. From the multivariate logistic regression analysis, predictors of checking the skin were: experienced tick bites in the past (OR 2.19; 95% CI 1.27-3.78), higher knowledge levels (OR 2.83; 95% CI 1.74-4.58) and higher levels of concern about LD (OR 2.81; 95% CI 1.71-4.60).

**Table 2:** Predictors of wearing protective clothing to prevent tick bites

**Characteristics**	**Wearing protective clothing (often/always)**						
	**Univariate**				**Multivariate**		
	**Row%**	**OR**	**95% CI**	**p-Value**	**OR**	**95% CI**	**p-Value**
**Employment status**							
employed	31	1.00			1.00		
unemployed/retired	46	1.96	1.27-3.03	0.002	1.96	1.24-3.08	0.004
**Knowledge**							
1-3 statements correct	33	1.00			1.00		
4-5 statements correct	46	1.80	1.15-2.81	0.01	1.69	1.07-2.68	0.03
**Feelings of concern**							
not (at all) worried (1-2)	28	1.00			1.00		
a bit/(very) worried (3-5)	45	2.07	1.34-3.21	0.001	2.22	1.41-3.51	0.001
**Perceived efficacy**							
certainly not, probably not, even (1-3)	17	1.00			1.00		
certainly/probably (4-5)	39	3.15	1.27-7.80	0.01	2.97	1.17-7.54	0.02

Gender, age, education, children in household, ethnicity, region of residence in the Netherlands, experienced tick bites in past, frequency of visiting nature, perceived severity (2 items) and perceived vulnerability (2 items) were not univariately associated with wearing protective clothing.

**Table 3:** Predictors of checking skin for the presence of ticks to prevent Lyme disease

**Characteristics**	**Checking skin (often/always)**						
	**Univariate**				**Multivariate**		
	**Row%**	**OR**	**95% CI**	**p-Value**	**OR**	**95% CI**	**p-Value**
**Experienced tick bites in the past**							
no, never	27	1.00			1.00		
yes, once/repeatedly/outside NL	48	2.48	1.48-4.14	0.001	2.19	1.27-3.78	0.005
**Knowledge**							
1-3 statements correct	24	1.00			1.00		
4-5 statements correct	47	2.89	1.82-4.59	<0.001	2.83	1.74-4.58	<0.001
**Perceived susceptibility**							
not (at all) susceptible (1-3)	25	1.00					
(very) susceptible (4-5)	44	2.34	1.48-3.70	<0.001	**-**	**-**	**-**
**Likelihood being diagnosed with Lyme disease in next 12 month**							
not (at all) likely (1-2)	25	1.00					
a bit/(very) likely (3-5)	39	1.92	1.23-3.01	0.004	**-**	**-**	**-**
**Feelings of concern**							
not (at all) worried (1-2)	20	1.00			1.00		
a bit/(very) worried (3-5)	43	3.04	1.89-4.90	<0.001	2.81	1.71-4.60	<0.001

Gender, age, education, employment status, children in household, ethnicity, region of residence, frequency of visiting nature, perceived severity of Lyme disease (2 items) and perceived efficacy were not univariately associated with checking skin.

### Main motivators and barriers

Respondents were asked to identify the main motivators and barriers for wearing protective clothing, using insect repellent skin products and checking skin/removing ticks from their skin (Table 4). Overall, the main motivators that were mentioned were: the perceptions that LD can be severe, the perception that the preventive measure is effective in preventing tick bite/LD, a person’s feeling of responsibility regarding his/her health and the perception that there is a high chance of tick bites. Among the 78 respondents who did not wear protective clothing, 81% reported that as a barrier, wearing protective clothing in summer is too warm; 30% reported a low risk of tick bites and 23% perceived that wearing protective clothing in nature areas is overdone. Of the 276 respondents who did not use insect repellent skin products, 34% did not believe these products to be effective, 32% did not like to use insect repellent products on their skin and 27% reported that too little information is provided about preventing tick bites through insect repellent skin products. Of the 108 respondents who did not check their skin/remove tick, 35% perceived low risk of tick bites, 19% did not know how to recognize a tick, 19% thought checking the skin after being outdoors is overdone, and 16% did not know how to remove a tick or reported that too little information is provided.

**Table 4:** Main motivators and barriers for measures to prevent tick bites

**Wearing protective clothing**	%
Motivators (n = 284)	
“wearing protective clothing is effective”	53
“high perceived chance of tick bites”	47
“Lyme disease can be severe”	37
“feel responsible for my health”	33
“I follow the advice”	19
Barriers (n = 78)	
“wearing protective clothing during summer is too warm”	81
“low perceived chance of tick bites”	30
“wearing protective clothing in nature is overdone”	23
“low perceived chance of Lyme disease”	19
**Using insect repellent skin products**	
Motivators (n = 86)	
“Lyme disease can be severe”	45
“feel responsible for my health”	40
“high perceived chance of tick bites”	38
“using repellent skin products is effective”	34
“I follow the advice”	29
Barriers (n = 276)	
“do not belief it is effective”	34
“do not like to use insect repellent products for my skin”	32
“too little information is provided”	27
“low perceived chance of tick bites”	23
“using insect repellent skin products is overdone”	22
“I am not familiar with insect repellent skin products”	19
**Checking skin after being outdoors/remove tick**	
Motivators (n = 353)	
“Lyme disease can be severe”	64
“checking skin/remove tick is effective”	48
“feel responsible for my health”	44
“I follow the advice”	25
“high perceived chance of tick bites”	18
“high perceived chance of Lyme disease”	16
Barriers (n = 108)	
“low perceived chance of tick bites”	35
“do not know how to recognize a tick”	19
“check my skin after being outdoors is overdone”	19
“do not know how to remove a tick”	16
“too little information is provided”	16

Reasons reported by <15% of the respondents are not included in this table.

## Discussion

In this study we identified the main predictors and motivators that influence protective behavior for preventing tick bites and LD. We did this by investigating the knowledge, perceptions and efficacy beliefs of healthy people in the general population in the Netherlands, a country in which the incidence of LD has increased sharply throughout the past two decades.

Insight into public perceptions and protective behavior regarding LD is crucial in order to develop a successful prevention program [9]. We conclude that good general knowledge about preventing tick bites and LD is scarce, while the perception of risks and self-efficacy of the measures varies greatly among the respondents.

Only 35% of the respondents reported a good general knowledge of LD, and only a quarter were aware that using repellent skin products can protect against tick bites. Suboptimal public knowledge regarding LD was also found in other studies in endemic areas. For example, Heller et al conducted a questionnaire study among 103 Brazilian residents -living in a Lyme disease endemic area in the United States-, and reported that 36% of the respondents had never heard of the disease and 62% were not certain they could recognize the symptoms [9]. Higher levels of knowledge seem to positively influence protective behavior as demonstrated by Gould et al. [10]. However, research in areas where LD is endemic has demonstrated that despite adequate knowledge about its symptoms and transmission, many people do not perform behaviors to reduce their risk of infection [25]. These findings suggest that a lack of knowledge is not the only reason for poor uptake of protective behavior.

Nearly all respondents perceived high severity of LD, but perceived vulnerability and feelings of anxiety were lower. The fact that the majority of the respondents perceived low personal risk of LD, implicates some public underestimation, especially, given the fact that people in the Netherlands, in particular those who often visit woodland areas, have a real risk of getting tick bites and developing LD [26]. The underestimation of risk is found to have been caused by factors such as lack of knowledge. Furthermore, if people underestimate their personal risk they will be less willing to engage in preventive behavior [13,27].

Higher levels of self-efficacy, respons efficacy and intention were observed for checking the skin after being outdoors and removing ticks if necessary. However, lower levels of (self-)efficacy and intention were observed for wearing protective clothing and using insect repellent skin products. The fact that most respondents in our study were unaware that using repellent skin products can protect against tick bites, might also be related to the lower levels of intention to use these products.

The percentage of respondents taking preventive measures ranged from 6% for using insect repellent skin products, to 40% for wearing protective clothing. These percentages are rather low, compared to other studies. Studies in the US reported that 66%-99% of the respondents took measures to prevent LD [10,22,23]. Furthermore, Heller et al found that the majority (78%) of the Brazilian respondents wore long trousers when outdoors and Herrington reported that one-half of the US respondents also did this [9,24]. The lower levels of wearing protective clothing in the Netherlands, especially in the summer, could be caused by the climate. The Netherlands has a maritime climate, with cool summers and an average temperature of 19 °C in July. People in the Netherlands like wearing (light) clothing, such as shorts and short sleeved shirts, if the temperature increases. Also the fact that people believe that wearing protective clothing in nature areas is overdone, as reported in this study, might be a reason for the low levels of wearing protective clothing as reported by Cartter et al. [13].

One-third of the respondents in our study reported checking their skin after being outdoors. This is comparable with other studies; i.e. Heller et al who described that only 28% of the Brazilian population check their skins for ticks [9]. The main barriers for checking skin for ticks reported in our study were low perceived personal risk and not knowing how to recognize a tick.

Only 6% of our respondents reported using repellent skin products. The low use of insect repellent skin products was also been found in other studies. For example, in Brazil and the US 66% and 69% of the respondents respectively never used insect repellent skin products for protection against LD [9]. In our study a barrier for using repellent skin products is that people are not convinced about their efficacy or do not like to use these products. Herrington investigated barriers for using insect repellent skin products, and found that a substantial proportion of US respondents believed that using insect repellent could make them ill [24]. This underlines the need for people to “believe” in the effectiveness of a recommended behavior as well as they should have appropriate knowledge on the subject.

There were some differences in public perceptions regarding LD among socio-demographic subgroups. For example, females reported higher levels of perceived efficacy and self-efficacy to check their skin after being outdoors, whereas older respondents (≥ 50 yrs) reported higher levels of perceived efficacy, self-efficacy and an intention to wear protective clothing. However, in multivariate analysis, of all socio-demographic variables only employment status remained a significant predictor for wearing protective clothing for preventing tick bites.

As reported in our study, having had tick bites in the past, higher levels of knowledge and moderate/high levels of concern were significant predictors for checking the skin. Significant predictors of wearing protective clothing were being unemployed/retired, higher knowledge levels, higher levels of concern about LD and higher levels of perceived efficacy of wearing protective clothing. These findings are in accordance with Herrington [24], reporting that having seen ticks, being concerned about being bitten, having heard about LD and knowing someone who had LD are the factors most predictive of specific tick-bite protective behavior. Mowbray et al. showed in his review that both knowledge and attitudes towards tick-borne disease are amenable to change via an education campaign [28]. Unfortunately, in his systematic review of all previous studies that assessed the impact of education or behavioral interventions on the uptake of behaviors intended to protect against tick-borne diseases he could find only nine studies, of which only three took the form of a randomized controlled trial (RCT) [28]. One RCT studied the willingness to the uptake of a vaccine for LD and two focussed on other protective measures. Lawless et all used an instructional video with a mock horror movie theme to improve knowledge, attitudes, and behaviors towards LD prevention in 13- to 16-year-olds from four Connecticut towns [29]. One month and six months after seeing the video, knowledge, attitudes and behavior had increased significantly in the intervention group. Another study investigating the effectiveness of an educational intervention was performed by Daltroy et al. In over 30000 passengers on ferry boats to a Lyme-endemic area of Nantucket Island. In this study controls received education about bike safety, while intervention participants received information on preventing tick-borne disease, particularly LD. Information was delivered on board by entertainers to make the messages more compelling. Two months after the intervention, experimental participants were more likely than controls to adopt precautionary behaviors, as well as to check themselves daily for ticks. In conclusion, future prevention programs for LD should focus on improving public knowledge, i.e. with regard to disease severity and vulnerability, efficacy of measures and on how to take preventive measures.

This is the first national study to evaluate the perceived LD-risk and protective behavior for LD in the general public in the Netherlands. Nevertheless, there are a number of limitations to our study. The majority of respondents (51%) was older than 50 years. This may have limited the generalizability of the results, although older age has not been found to be a distinct factor associated with compliance to preventive measures for LD found in previous studies. Furthermore, potential selection bias may have been introduced in that only respondents with a computer were interviewed by this online survey. Finally, cross sectional studies can prove a rich baseline of data points but should not be used to make causal statements, given the lack of a temporal sequence of events.

## Conclusion

Our study has several implications for the development of LD prevention programs. It demonstrates that knowledge, level of concern and perceived efficacy of measures are the main determinants of preventive behavior. Therefore future prevention programs should focus on these determinants, for example, by providing facts and raising awareness about LD and protective measures that can be taken. Since protective measures like wearing protective clothing and using insect repellents are not ‘popular’, it is important that prevention programs focus on removing any barriers for complying with these protective measures, especially in people who have never had tick bites and those who are less concerned about the risks. Furthermore, it is important to tailor the information to specific socio-demographic subgroups and high risk groups.

Promoting preventive measures for LD is really important since reducing the tick population and developing a vaccine can only be seen as long-term solutions for the problems. The results of this study can be used as a base for developing effective prevention programs that connect with the needs of the target group, with the main goal to increase compliance with recommended measures.

The challenge is to take the principles demonstrated in this study and apply them to prevention programs. Some work in this area has already been done. Last year the Netherlands National Institute of Public Health and The Environment redesigned the national public information campaign on ticks and LD in the Netherlands. In this campaign the focus was shifted. First, not all possible evidence based preventive measures on LD were presented in the communication, instead the focus was placed on checking the skin and removing ticks. Also a educational game was developed, called in Dutch: “Teekcontrol.nl“, to teach children playfully about ticks and LD. In this online game children can discover where ticks are most likely to be found and why it is important to check the skin after playing outdoors. Within 8 months of launching over 30.000 children had played this game. The learning effect of the game will be evaluated in 2012. Furthermore, a mobile phone app on ticks and LD is currently being developed. This will be based on a user- centered design. This means that the public will determine the features of this application.

### Consent

Written informed consent was obtained from the patient for publication of this report and any accompanying images.

## Competing interests

The authors declare that they have no competing interests.

## Authors’ contribution

All authors contributed to the study design. DB, MB, and HV played major roles in the data collection process. Data analysis was performed by MB and HV with advice from DB and JvS. DB, MB and JvS wrote the first draft of the manuscript; HV critiqued the manuscript and contributed to further drafts. All authors read and approved the final manuscript.

## Pre-publication history

The pre-publication history for this paper can be accessed here:

http://www.biomedcentral.com/1471-2458/13/225/prepub

## References

[B1] Hofhuis A Harms MG Van der Giessen JWB Sprong H Notermans DW van Pelt W Ziekte van Lyme in Nederland 1994-2009 Infectieziektenbulletin 2010 21 84 87

[B2] Smith R Takkinen J Editorial Team Lyme borreliosis: Europe wide coordinated surveillance and action needed? Eurosurveillance 2006 25 2 16819127 10.2807/esw.11.25.02977-en

[B3] Heyman P Cochez C Hofhuis A Van der Giessen J Sprong H Porter SR Losson B Saugerman C Donoso-Mantke O Niedrig M Papa A A clear present danger: tick-borne diseases in Europe Expert Rev Anti Infect Ther 2010 8 33 50 10.1586/eri.09.118 20014900

[B4] Gray JS The ecology of ticks transmitting Lyme borreliosis Exp Appl Acarol 1998 V22 249 258

[B5] Signs and Symptoms of Lyme Diseasehttp://www.cdc.gov/lyme/signs_symptoms/index.html

[B6] British Infection Association The epidemiology, prevention, investigation and treatment of Lyme borreliosis in Unites Kingdom patients: A position statement by the British Infection Association J Infect 2011 62 329 338 10.1016/j.jinf.2011.03.006 21421007

[B7] LCI (Coordinator Infectious Diseases Netherlands): LCI-guideline Lymeborreliose, May 2005 Available from: http://www.rivm.nl/richtlijnlyme

[B8] Poland GA Prevention of Lyme Disease: a review of the evidence Mayo Clin Proc 2001 76 13 24 10.4065/76.7.713 11444404

[B9] Heller JE Benito-Garcia E Maher NE Chibnik LB Maher CP Shadick NA Behavioral and attitudes survey about Lyme disease among a Brazilian population in the endemic area of Martha’s Vineyard, Massachusetts J Immigr Minor Health 2010 12 3 377 383 Epub 2008 Sep 16 10.1007/s10903-008-9187-6 18792780

[B10] Hannah GL Nelson Randall S Griffith Kevin S Hayes Edward B Piesman J Mead Paul S Cartter Mathhew L Knowledge, attitudes, and behaviors regarding Lyme disease prevention among Connecticut residents, 1999-2004 Vector Borne Zoonotic Dis 2008 8 6 769 776 PMID 18637724 10.1089/vbz.2007.0221 18637724

[B11] Brewer NT Weinstein ND Cuite CL Herrington JE Risk perceptions and their relation to risk behavior Ann Behav Med 2004 27 2 125 130 10.1207/s15324796abm2702_7 15026296

[B12] Centers for Disease Control (CDC) Lyme disease knowledge, attitudes, and behaviors- Connecticut MMWR Morb Mortal Wkly Rep 1992 41 28 505 507 1630426

[B13] Cartter ML Farley TA Ardito HA Hadler JL Lyme disease prevention–knowledge, beliefs, and behaviors among high school students in an endemic area Conn Med 1989 53 6 354 356 2758823

[B14] Kok G Planmatige ontwikkeling van op theorieën en evidentie gebaseerde gezondheidsbevordering, met als voorbeeld de ziekte van Lyme Infectieziektenbulletin 2006 17 3 102 103

[B15] Maat A Konings F **Teek it or leave it? Onderzoek van GGD West-Brabant naar preventieve maatregelen tegen tekenbeten** Infectieziektenbulletin 2010 7 221 223 in Dutch, available from: http://www.rivm.nl/teekit

[B16] Norman P Boer H Seydel ER Conner M, Norman P Protection Motivation Theory Predicting health behaviour 2005 Berkshire. UK: Open University Press 81 126

[B17] Champion VL Skinner CS Glanz K, Rimer BK, Viswanath K The Health Belief Model Health behavior and health education: theory, research, and practice 2008 San Francisco. CA: Jossey Bass 45 65

[B18] Central Committee on Research involving Human Subjects Manual for the review of medical research involving human subjectshttp://www.ccmo-online.nl

[B19] Bults M Beaujean DJMA de Zwart O Kok G van Empelen P van Steenbergen JE Perceived risk, anxiety, and behavioural responses of the general public during the early phase of the Influenza A (H1N1) pandemic in the Netherlands: results of three consecutive online surveys BMC Publ Health 2011 11 2 10.1186/1471-2458-11-2 PMC3091536 21199571

[B20] Brug J Aro AR Oenema A de Zwart O Richardus JH Bisschop GD SARS risk perception, knowledge, precautions, and information sources, the Netherlands Emerg Infect Dis 2004 10L 1486 1489 15496256 10.3201/eid1008.040283 PMC3320399

[B21] de Zwart O Veldhuijzen IK Elam G Aro AR Abraham T Bishop GD Richardus JH Brug J **Avian influenza riks perception, Europe and Asia.** Emerg Infect Dis 2007 13 290 293 10.3201/eid1302.060303 17479894 PMC2725846

[B22] Phillips CB Liang MH Sangha O Wright EA Fossel AH Lew RA Fossel KK Shadick NA Lyme disease and preventive behaviors in residents of Nantucket Island, Massachusetts Am J Prev Med 2001 20 3 219 224 10.1016/S0749-3797(00)00315-9 11275450

[B23] Shadick NA Daltroy LH Phillips CB Liang US Liang MH Determinants of tick-avoidance behaviors in an endemic area for Lyme disease Am J Prev Med 1997 13 4 265 270 9236962

[B24] Herrington JE Risk perceptions regarding ticks and Lyme Disease: a national survey Am J Prev Med 2004 26 135 140 10.1016/j.amepre.2003.10.010 14751325

[B25] Corapi KM White MI Phillips CB Strategies for primary and secondary prevention of Lyme disease Nature Clinical Practice 2007 3 20 25 10.1038/ncprheum0374 17203005

[B26] de Mik EL van Pelt W Docters-van Leeuwen B The geographical distribution of tick bites and erythema migrans in general practice in the Netherlands Int J Epidemiol 1997 26 451 457 10.1093/ije/26.2.451 9169184

[B27] Slovic P Perception of risk Science 1987 236 280 285 10.1126/science.3563507 3563507

[B28] Mowbray F Amlot R Rubin GJ Ticking all the boxes? A systematic review of education and communication interventions to prevent tick-borne disease Vector Borne Zoonotic Dis 2012 18 1 9 10.1089/vbz.2011.0774 PMC3438805 22607072

[B29] Laweless KA Brown SW Cartter M Applying educational psychology and instructional technology to health care issues: Combating Lyme disease Int J Instr Media 1997 24 287 297

